# Correction: Novel pyrimidine-substituted chalcones: In vitro antioxidant properties and cytotoxic effects against human cancer cell lines

**DOI:** 10.1371/journal.pone.0344376

**Published:** 2026-03-05

**Authors:** Prashant Nayak, Vikram S. Shenoy, Vijay Upadhye, Kasim Sakran Abass, Omar Awad Alsaidan, Mukesh Soni, Sunil Tulshiram Hajare

There is an error in affiliation 1 for author Prashant Nayak. The correct affiliation 1 is: Nitte (Deemed to be University), NGSM Institute of Pharmaceutical Sciences, Department of Pharmaceutics, Mangaluru-575018, Karnataka, India.
