# Supplementary figures and images for: Novel pyrimidine-substituted chalcones: In vitro antioxidant properties and cytotoxic effects against human cancer cell lines
Source: PLoS One. 2025 Nov 3;20(11):e0334620. doi: 10.1371/journal.pone.0334620 (PMC12582495; doi:10.1371/journal.pone.0334620)

Figure S1. Simulated  $^1\text{H}$  NMR Spectrum of CFMPY-2 (400 MHz,  $\text{DMSO-d}_6$ )

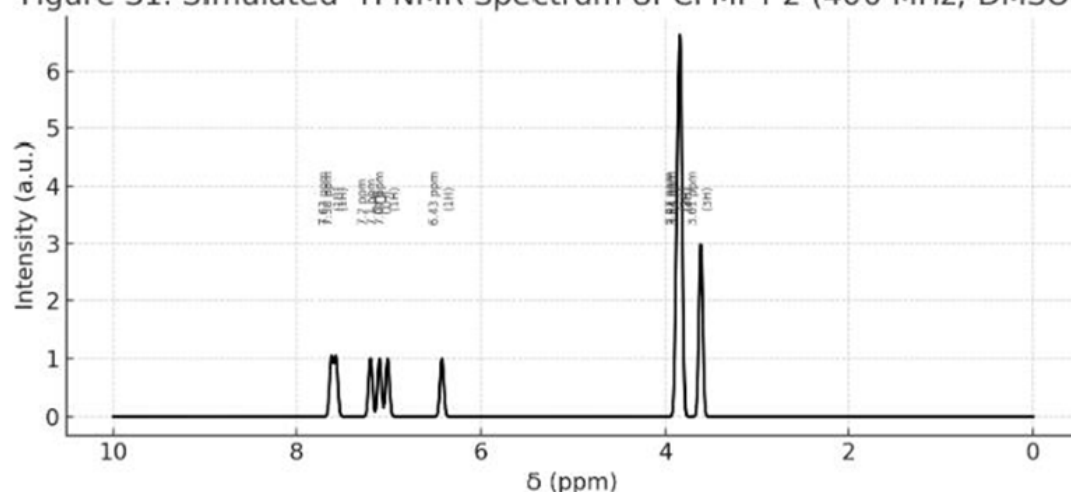

Supplement: S1 Fig — Shows aromatic and methoxy proton regions confirming the structure. (PDF) [file pone.0334620.s001.pdf]

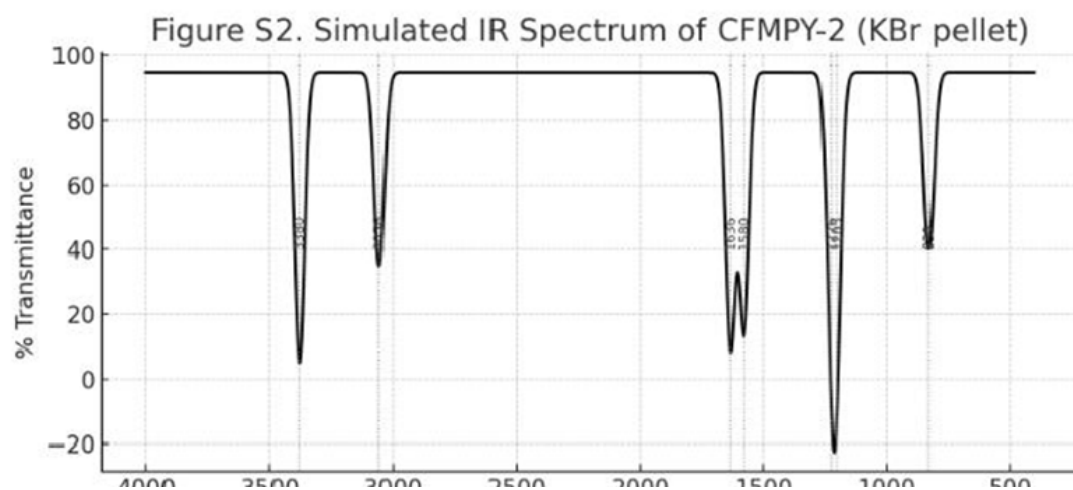

Supplement: S2 Fig — Indicates methoxy and aromatic proton patterns supporting substitution. (PDF) [file pone.0334620.s002.pdf]

Figure S3. Simulated MS Spectrum of CFMPY-2

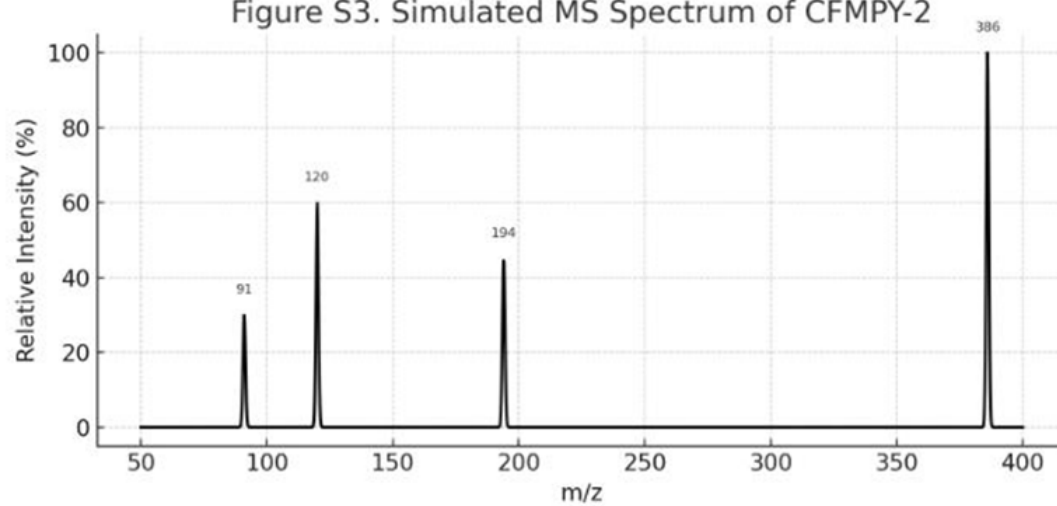

Supplement: S3 Fig — Displays characteristic proton splitting of substituted phenyl ring. (PDF) [file pone.0334620.s003.pdf]

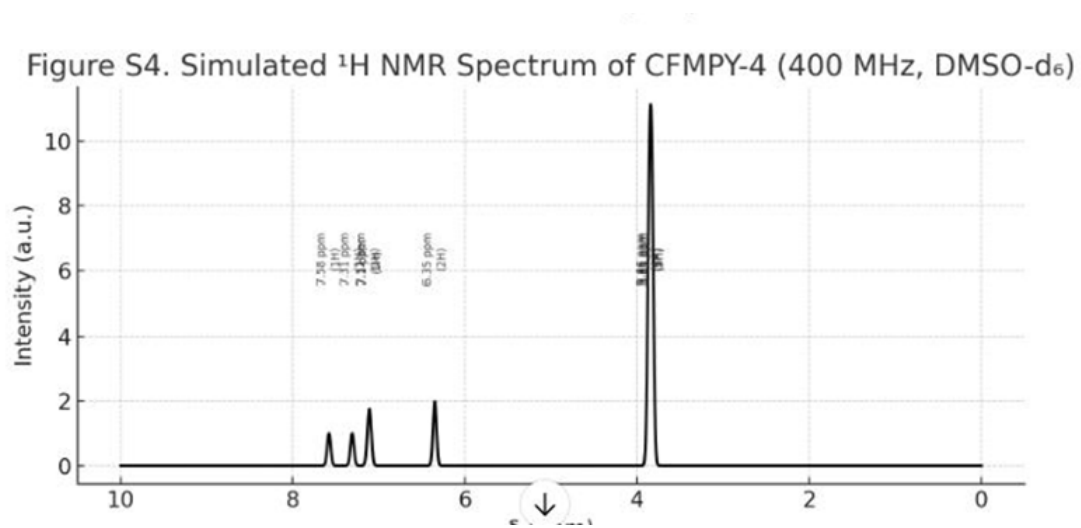

Supplement: S4 Fig — Shows defined peaks consistent with methoxy-substituted pyrimidine. (PDF) [file pone.0334620.s004.pdf]

Figure S5. Simulated IR Spectrum of CFMPY-4 (KBr pellet)

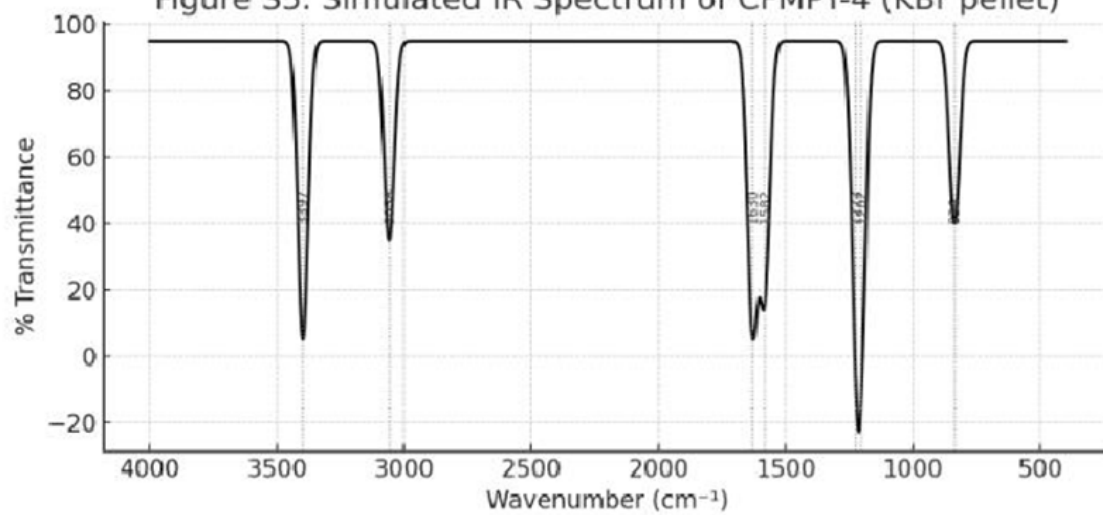

Supplement: S5 Fig — Depicts proton signals for methoxy and aromatic regions. (PDF) [file pone.0334620.s005.pdf]

Figure S6. Simulated MS Spectrum of CFMPY-4

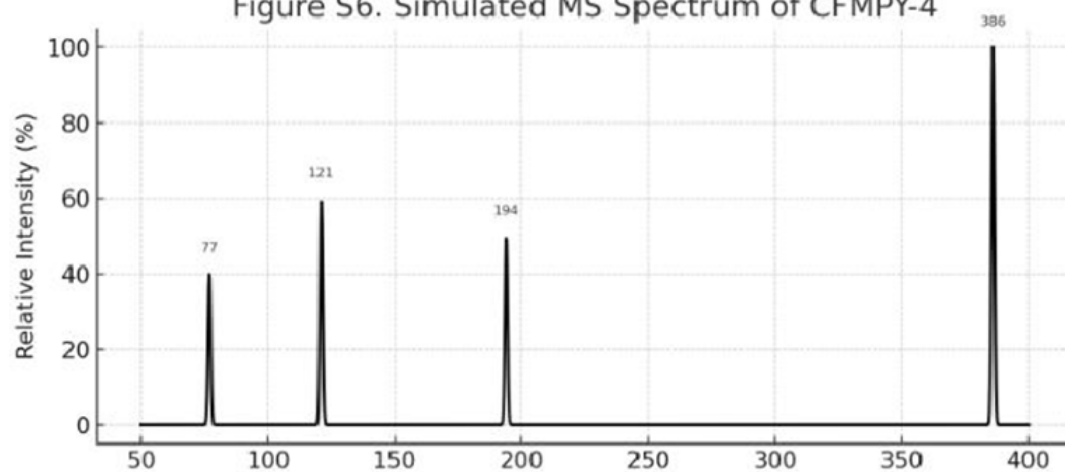

Supplement: S6 Fig — Highlights specific downfield shifts corresponding to electron-rich substituents. (PDF) [file pone.0334620.s006.pdf]

Figure S7. Simulated  $^1\text{H}$  NMR Spectrum of CFMPY-15 (400 MHz,  $\text{DMSO-d}_6$ )

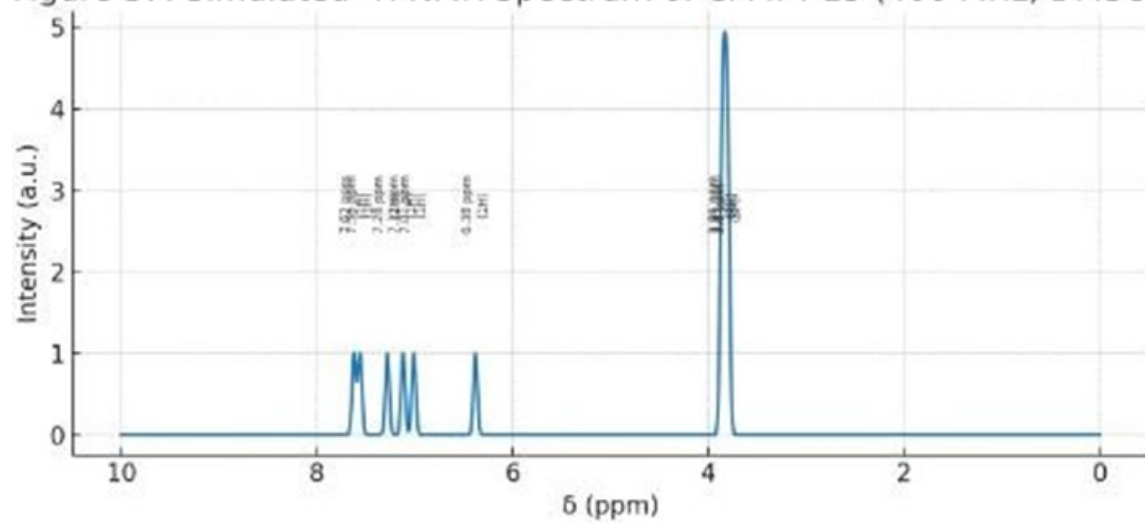

Supplement: S7 Fig — Focuses on methoxy group shifts and splitting pattern. (PDF) [file pone.0334620.s007.pdf]

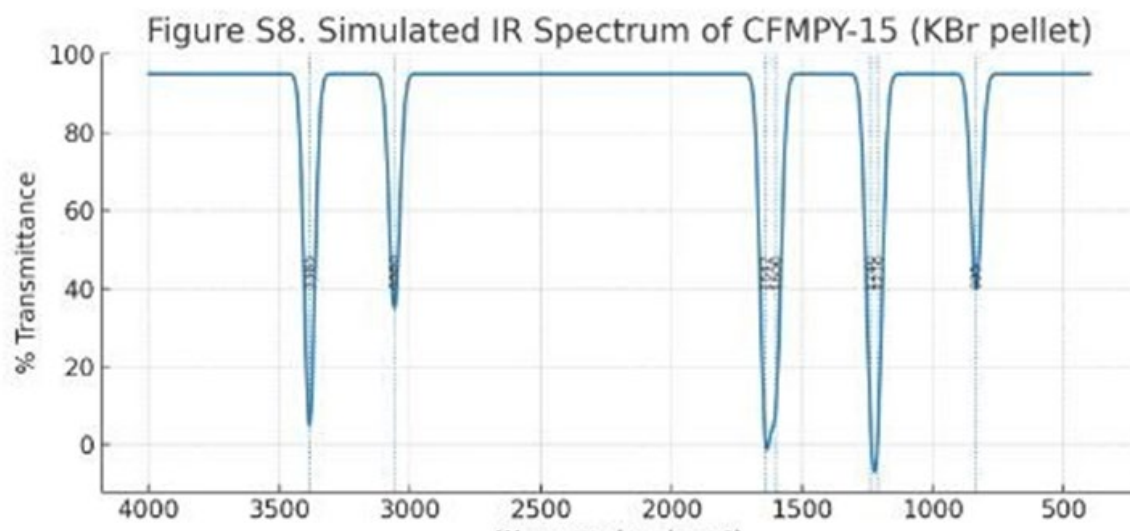

Supplement: S8 Fig — Emphasizes coupling constants and ring proton patterns. (PDF) [file pone.0334620.s008.pdf]

Figure S9. Simulated MS Spectrum of CEMPY-15

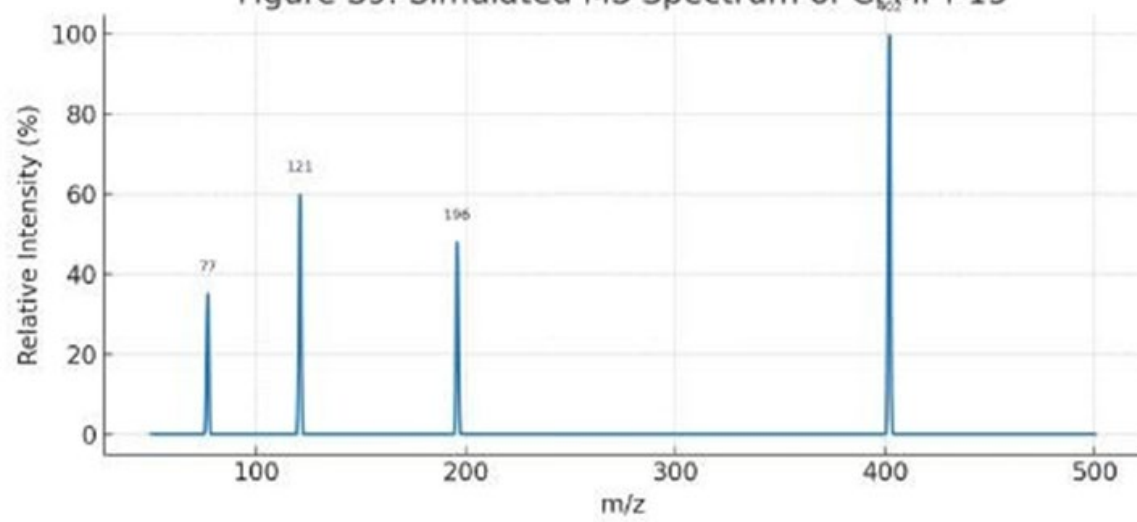

Supplement: S9 Fig — Clarifies overlapping regions in the aromatic range. (PDF) [file pone.0334620.s009.pdf]

Figure S10. Simulated  $^1\text{H}$  NMR Spectrum of CFMPY-17 (400 MHz,  $\text{DMSO-d}_6$ )

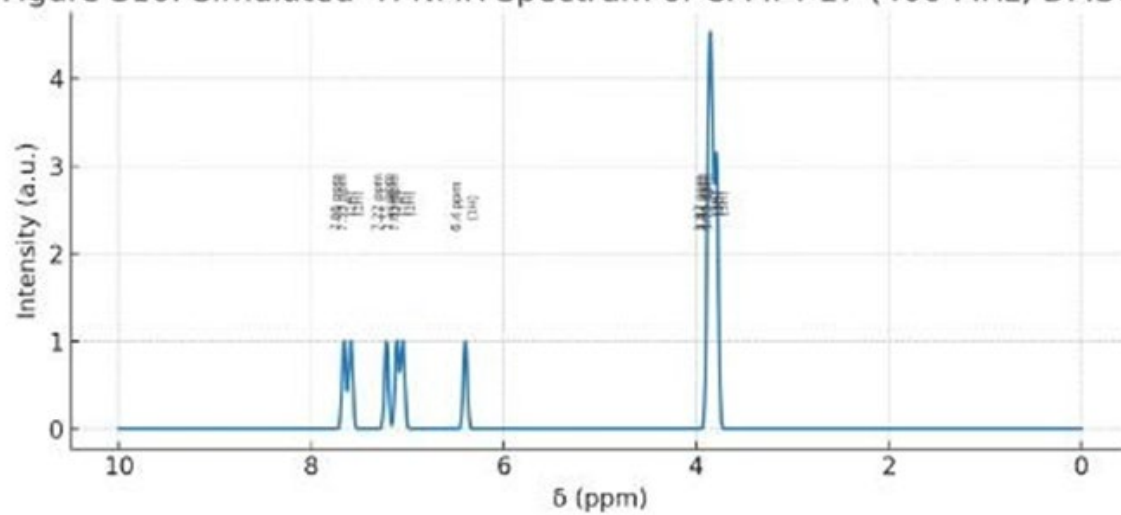

Supplement: S10 Fig — Displays proton environments around pyrimidine core. (PDF) [file pone.0334620.s010.pdf]

Figure S11. Simulated IR Spectrum of CFMPY-17 (KBr pellet)

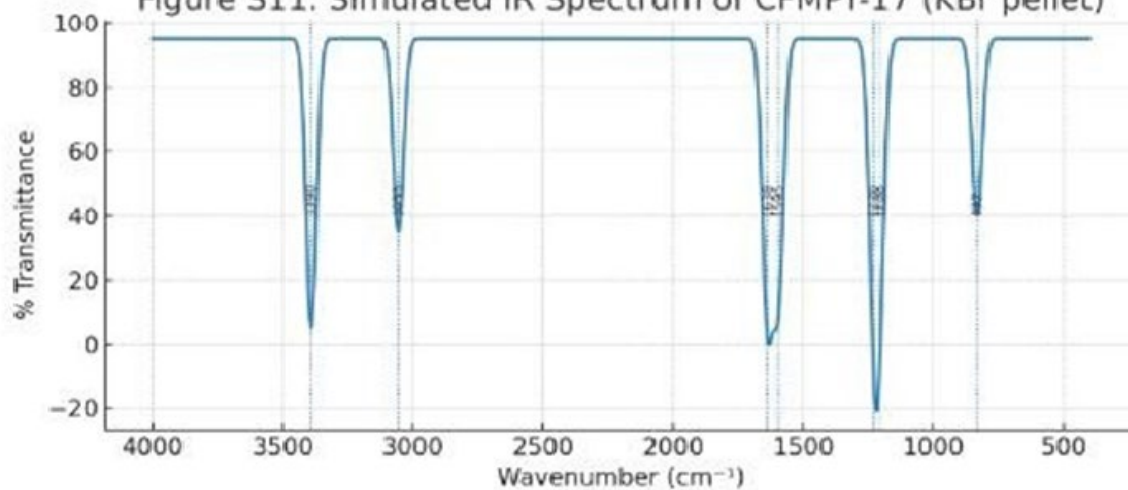

Supplement: S11 Fig — Shows cleaner resolution of overlapping signals. (PDF) [file pone.0334620.s011.pdf]

Figure S12. Simulated MS Spectrum of CFMPY-17

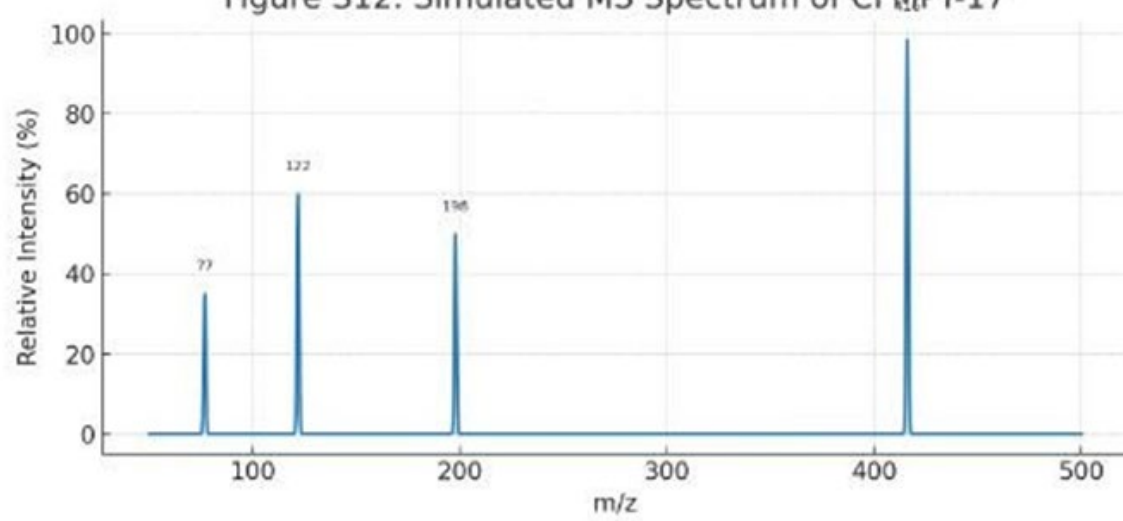

Supplement: S12 Fig — Further confirms signal integrity for aromatic protons. (PDF) [file pone.0334620.s012.pdf]

Figure S13. Simulated  $^1\text{H}$  NMR Spectrum of CFMPY-28 (400 MHz,  $\text{DMSO-d}_6$ )

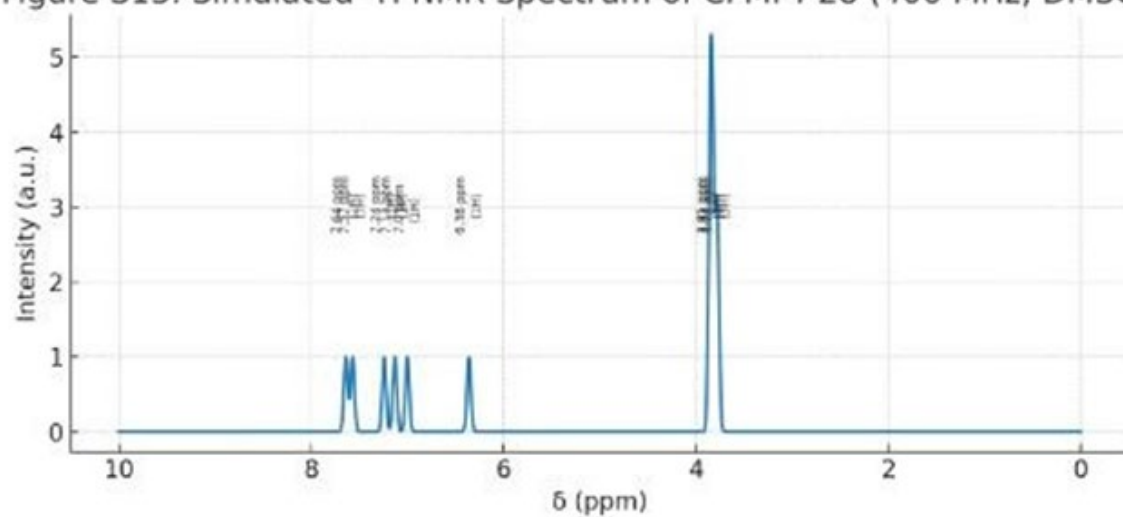

Supplement: S13 Fig — Enhances detail around methyl/methoxy substitutions. (PDF) [file pone.0334620.s013.pdf]

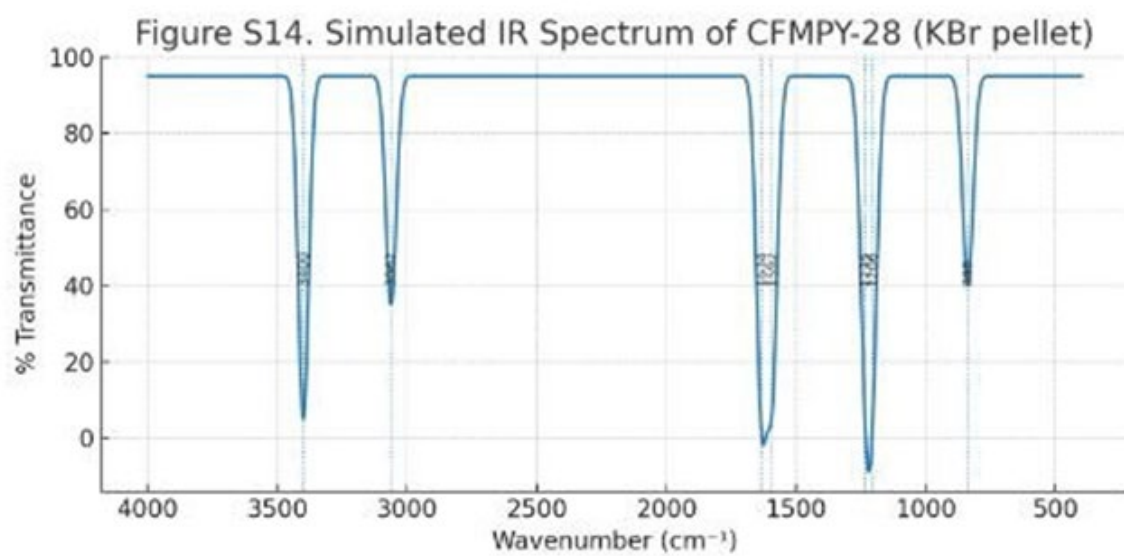

Figure S15. Simulated MS Spectrum of CFMPY-28

Supplement: S14 Fig — Confirms consistency of multiple methoxy group signals. (PDF) [file pone.0334620.s014.pdf]

Figure S15. Simulated MS Spectrum of CFMPY-28

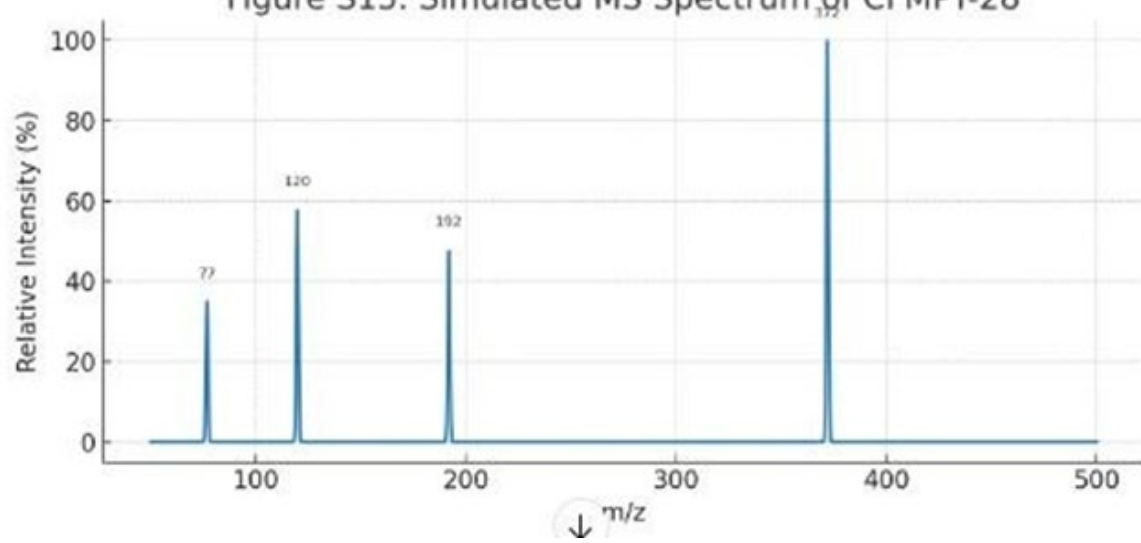

Supplement: S15 Fig — Distinguishes ring protons from side-chain environments. (PDF) [file pone.0334620.s015.pdf]

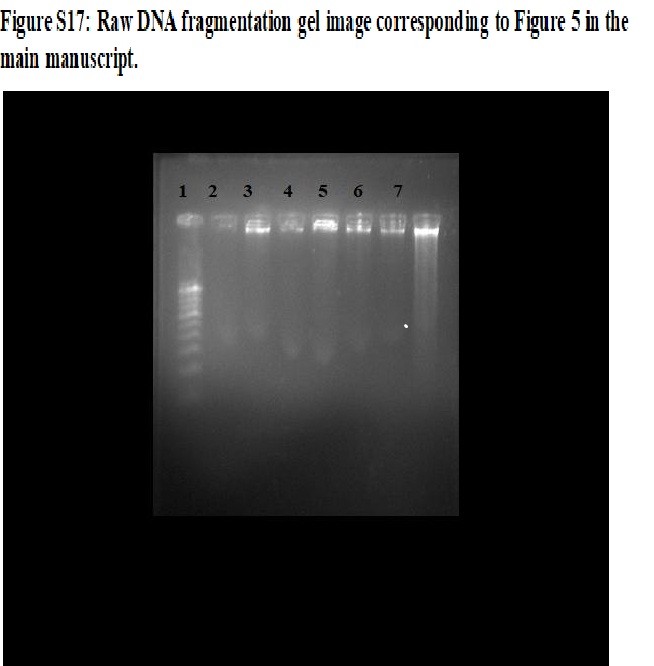

Supplement: S17 Fig — Original gel documentation showing DNA fragmentation patterns in HeLa cells treated with synthesized compounds. Lanes: 1 – DNA Ladder (molecular weight marker), 2 - CFMPY-2, 3 - CFMPY-4, 4 - CFMPY-15, 5 - CFMPY-17, 6 - CFMPY-28, 7 – Cisplatin (positive control). This uncropped image is provided for transparency and to demonstrate the absence of gel manipulati. (JPG) [file pone.0334620.s017.jpg]
