# Supplementary material for: Novel pyrimidine-substituted chalcones: In vitro antioxidant properties and cytotoxic effects against human cancer cell lines
Source: PLoS One. 2025 Nov 3;20(11):e0334620. doi: 10.1371/journal.pone.0334620 (PMC12582495; doi:10.1371/journal.pone.0334620)
Supplement: S16 Fig — Reference figure matching spectra with corresponding compound names and positions. (PDF) [file pone.0334620.s016.pdf]

Figure S16: Chemical structures on all NMR spectra

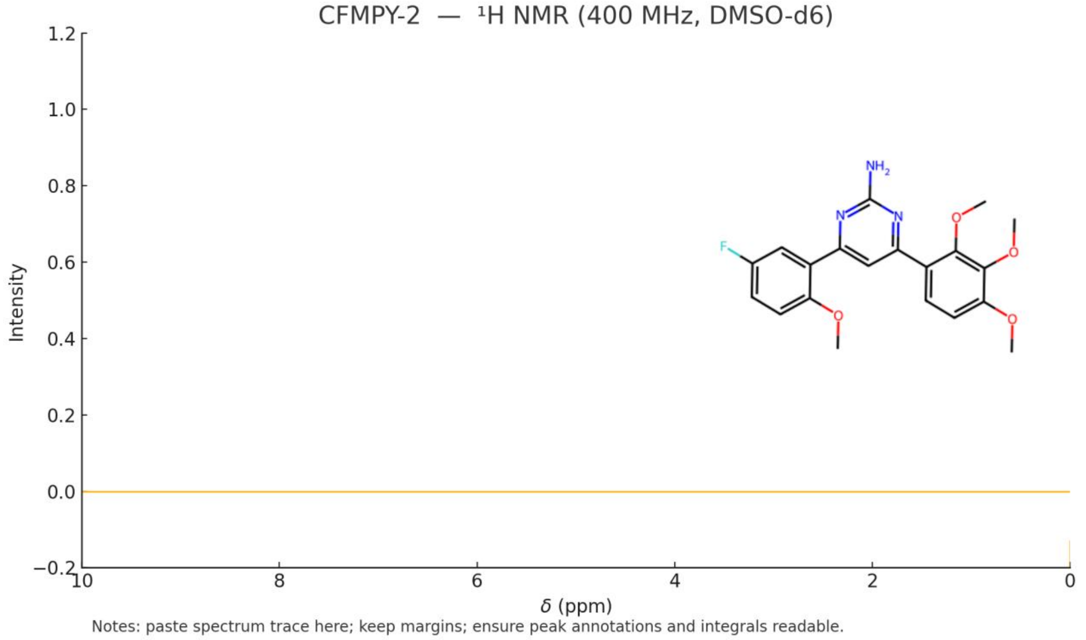

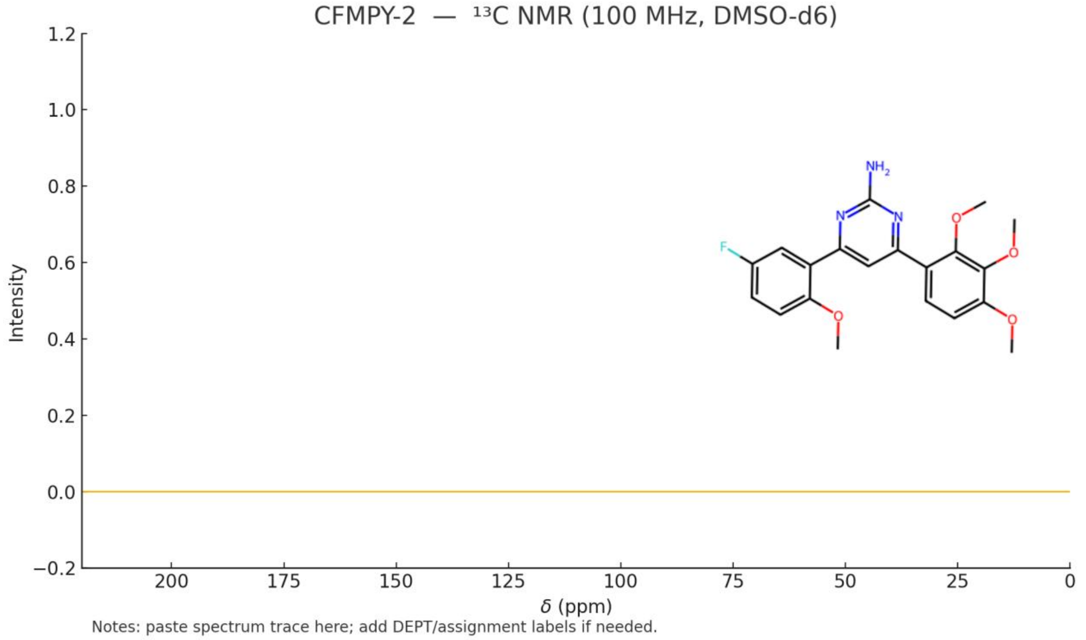

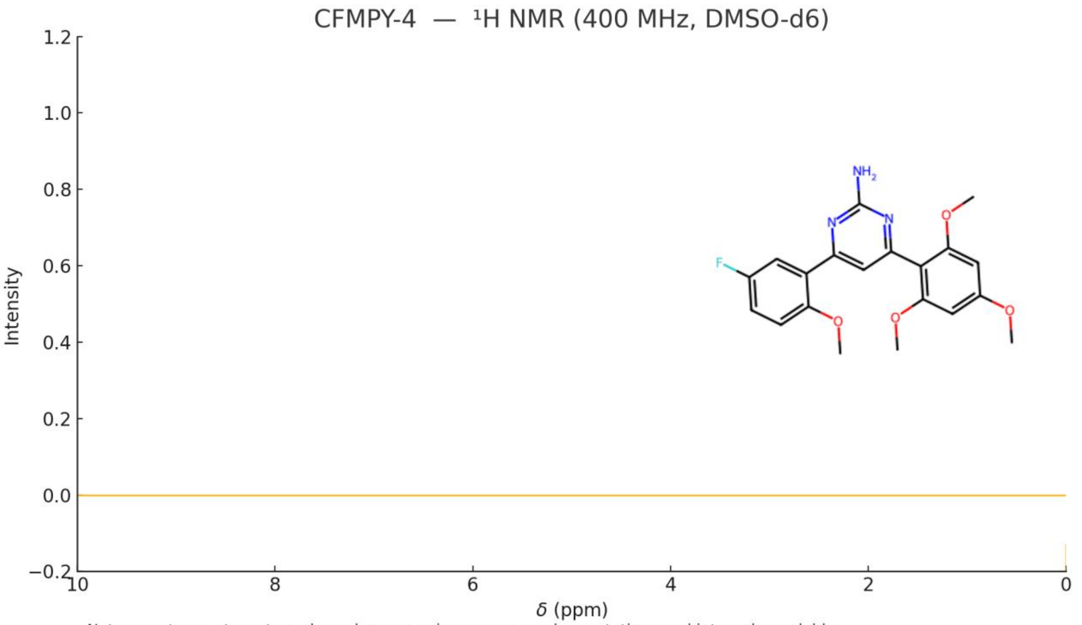

Notes: paste spectrum trace here; keep margins; ensure peak annotations and integrals readable.

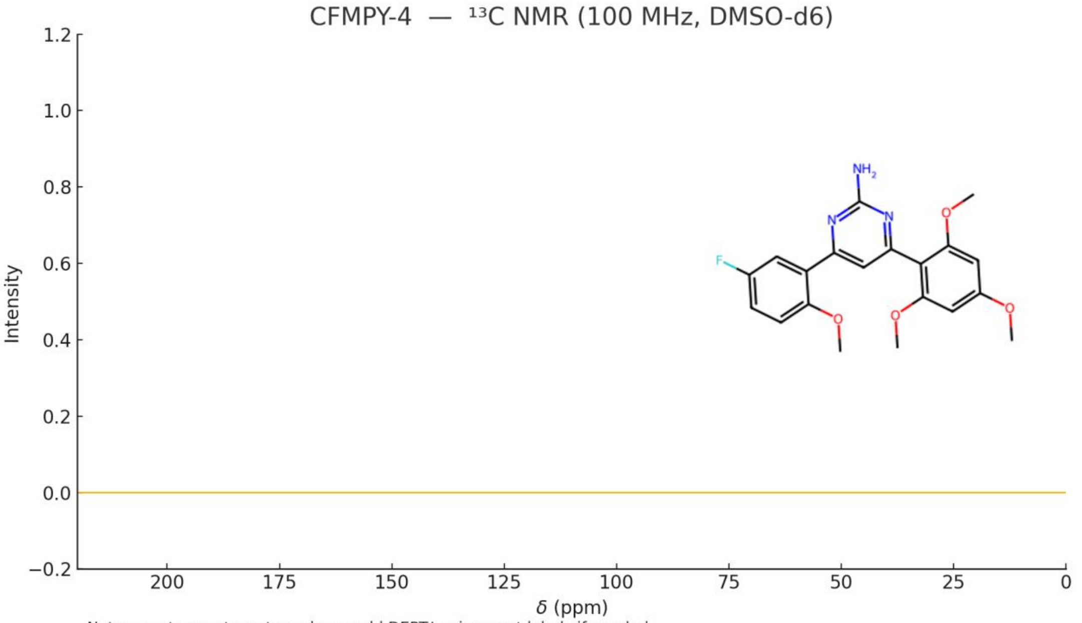

Notes: paste spectrum trace here; add DEPT/assignment labels if needed.

CFMPY-15 — ¹H NMR (400 MHz, DMSO-d6)

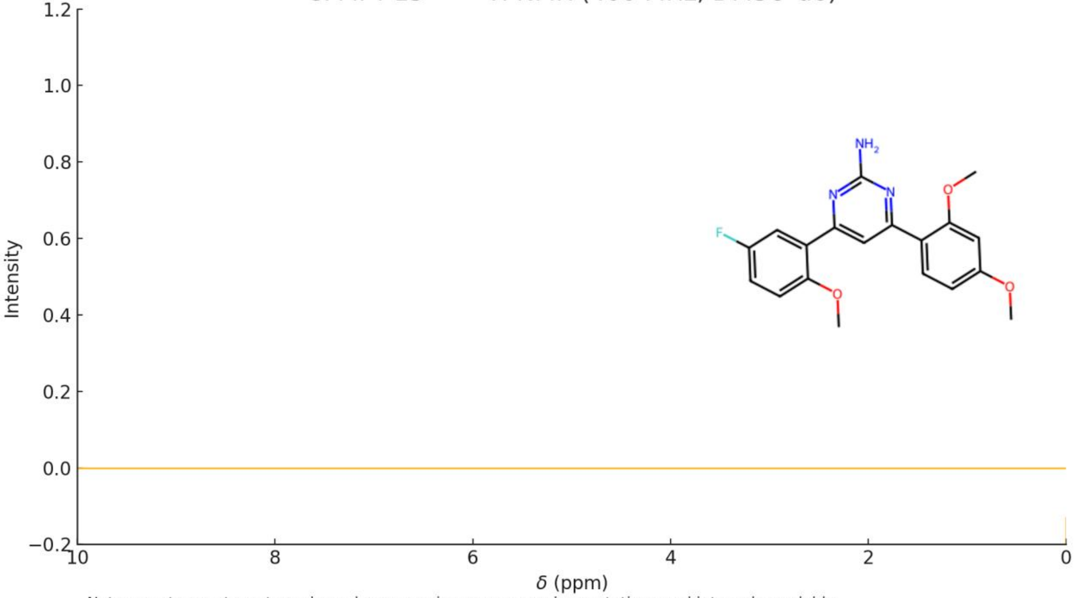

Notes: paste spectrum trace here; keep margins; ensure peak annotations and integrals readable.

CFMPY-15 — ¹³C NMR (100 MHz, DMSO-d6)

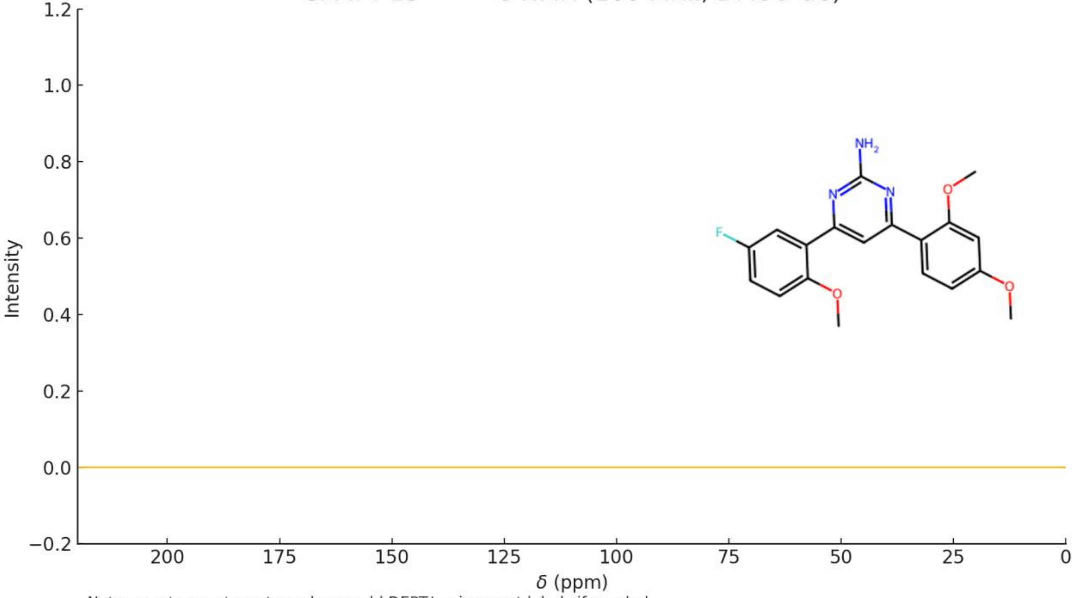

Notes: paste spectrum trace here; add DEPT/assignment labels if needed.

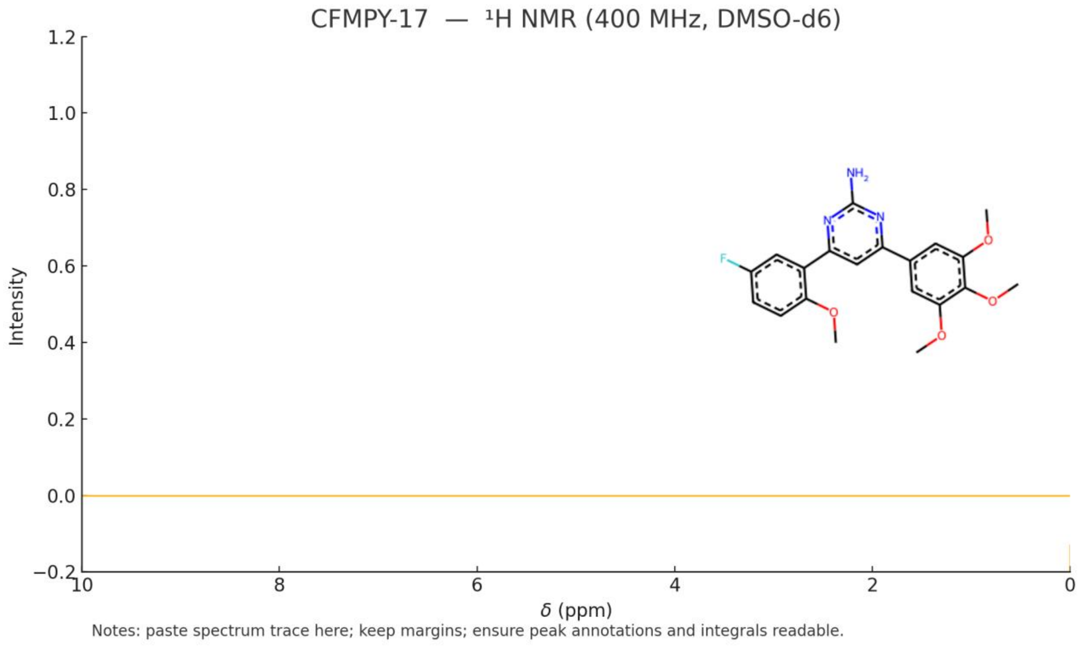
CFMPY-17 — ¹H NMR (400 MHz, DMSO-d6)
Intensity
1.2
1.0
0.8
0.6
0.4
0.2
0.0
−0.2
10
8
6
4
2
0
δ (ppm)
NH2
N
N
F
O
O
O
O
Notes: paste spectrum trace here; keep margins; ensure peak annotations and integrals readable.

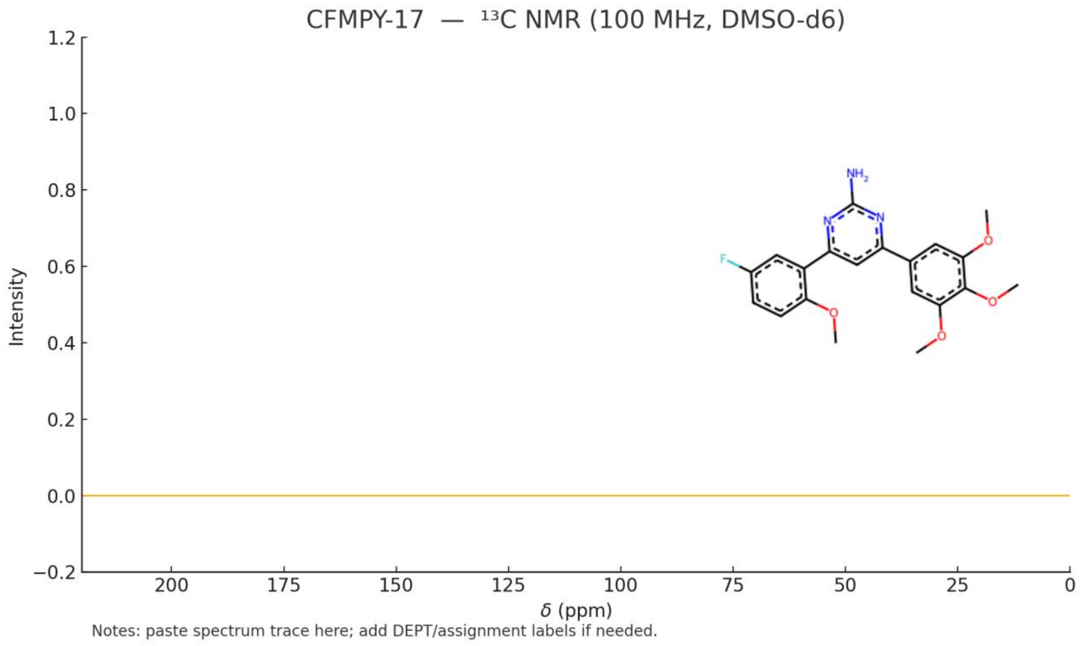
CFMPY-17 — ¹³C NMR (100 MHz, DMSO-d6)
Intensity
1.2
1.0
0.8
0.6
0.4
0.2
0.0
−0.2
200
175
150
125
100
75
50
25
0
δ (ppm)
NH2
N
N
F
O
O
O
O
Notes: paste spectrum trace here; add DEPT/assignment labels if needed.

CFMPY-28

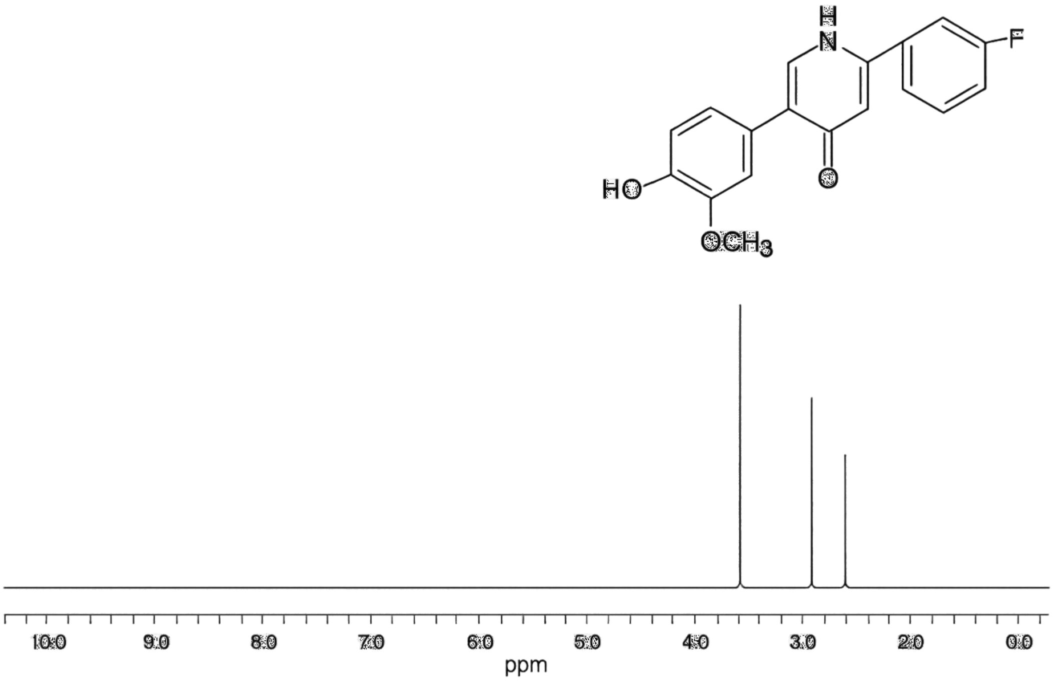
H
N
F
HO
O
OCH3
10.0
9.0
8.0
7.0
6.0
5.0
4.0
3.0
2.0
0.0
ppm

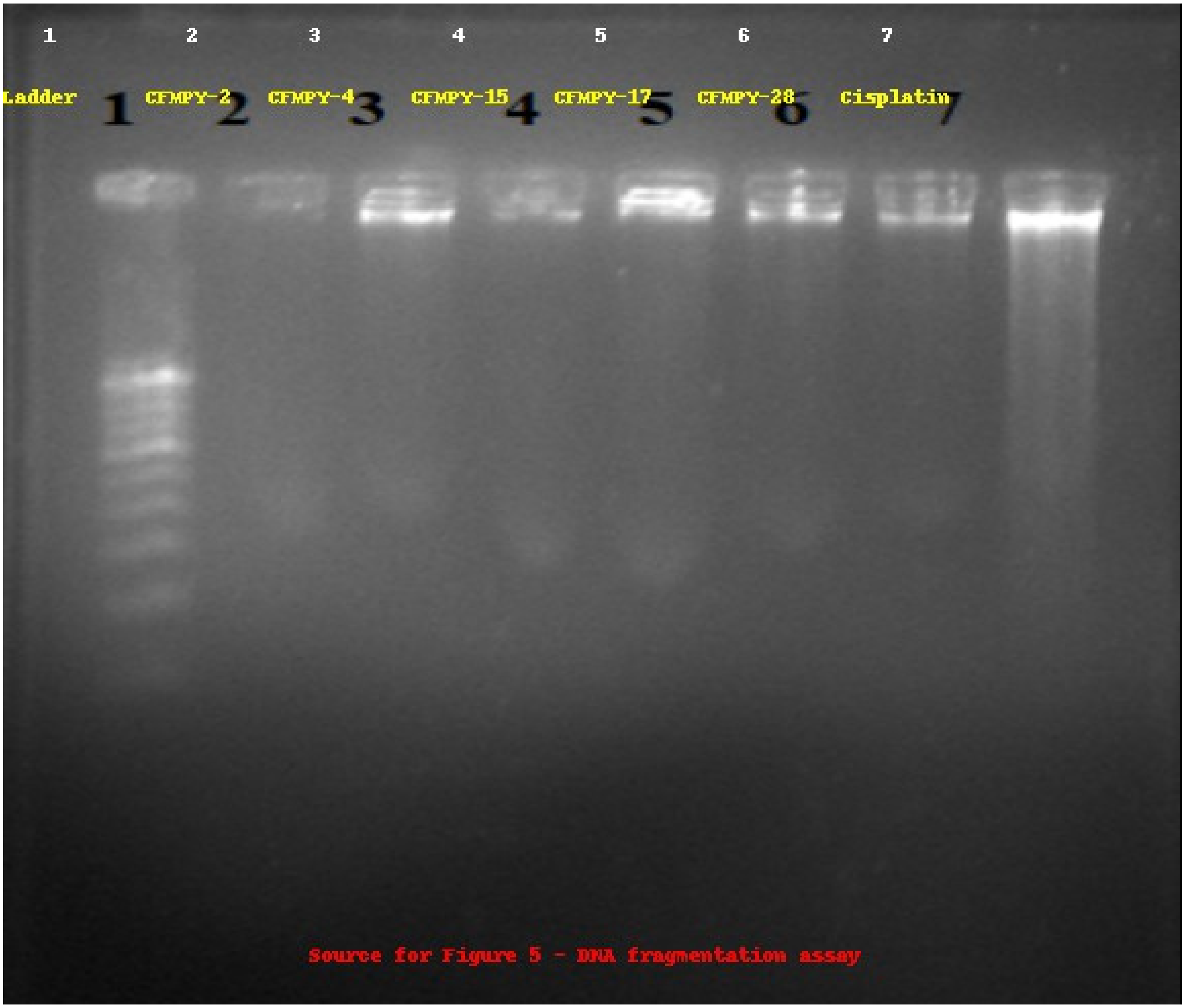

Figure S17. Raw DNA fragmentation gel image corresponding to Figure 5 in the main manuscript.
Lane 1: DNA Ladder; Lane 2: CFMPY-2; Lane 3: CFMPY-4; Lane 4: CFMPY-15; Lane 5: CFMPY-17; Lan
The uncropped gel image is provided with annotated lane identities and marker.
