# Supplementary material for: Novel pyrimidine-substituted chalcones: In vitro antioxidant properties and cytotoxic effects against human cancer cell lines
Source: PLoS One. 2025 Nov 3;20(11):e0334620. doi: 10.1371/journal.pone.0334620 (PMC12582495; doi:10.1371/journal.pone.0334620)
Supplement: Attachment — Submitted filename: Reviewing comments.docx [file pone.0334620.s018.docx]

Dear Editor,

I hope my recommendations finds you well,

Herein the recommendations of the manuscript "In Vitro Antioxidant and Anticytotoxic Activities of Synthesized Pyrimidine-Substituted Chalcones"

First, I want to declare that the supplementary data verification is the editor^’^s responsibility.

Reviewing comments:

1) I recommend updating the references of introduction to be mostly ranging from (2020-2025)

2) you need to enrich the introduction by adding a graphical abstract correlates the targeted nucleus and its modifications with the aimed anticancer activity of your work.

2) In the experimental section I recommend mentioning the molarity of the NaOH used in the preparation of Chalcones procedure.

3) Major revision: Experimental section:

All of the Synthesized Pyrimidine derivatives were written as pyridine-2 amine in the IUPAC name…not pyrimidine.
